# Clinical Advances in Molecular Biomarkers for Cancer Diagnosis and Therapy

**DOI:** 10.3390/ijms140714771

**Published:** 2013-07-16

**Authors:** Seema Sethi, Shadan Ali, Philip A. Philip, Fazlul H. Sarkar

**Affiliations:** 1 Department of Pathology, Karmanos Cancer Institute, Wayne State University School of Medicine, Detroit, MI 48201, USA; E-Mail: sseth@med.wayne.edu; 2 Department of Oncology, Karmanos Cancer Institute, Wayne State University School of Medicine, Detroit, Michigan, MI 48201, USA; E-Mails: alis@karmanos.org (S.A.); philipp@karmanos.org (P.A.P.)

**Keywords:** advances, cancer diagnosis, basic, clinical, translational research

## Abstract

Cancer diagnosis is currently undergoing a paradigm shift with the incorporation of molecular biomarkers as part of routine diagnostic panel. The molecular alteration ranges from those involving the DNA, RNA, microRNAs (miRNAs) and proteins. The miRNAs are recently discovered small non-coding endogenous single-stranded RNAs that critically regulates the development, invasion and metastasis of cancers. They are altered in cancers and have the potential to serve as diagnostic markers for cancer. Moreover, deregulating their activity offers novel cancer therapeutic approaches. The availability of high throughput techniques for the identification of altered cellular molecules allowed their use in cancer diagnosis. Their application to a variety of body specimens from blood to tissues has been helpful for appreciating their use in the clinical context. The development of innovative antibodies for immunohistochemical detection of proteins also assists in diagnosis and risk stratification. Overall, the novel cancer diagnostic tools have extended their application as prognostic risk factors and can be used as targets for personalized medicine.

## 1. Introduction

Recent years have seen a remarkable progress in the basic, translational and clinical research in cancers. This has led to scientific and technological advances which have opened new vistas for cancer diagnosis. National comprehensive cancer network (NCCN) reported tumor markers for six major malignancies in defining the optimum test that will help patient care [1]. Significant contributions in this aspect have been made by our group evaluating these molecular alterations in the pathogenesis of a wide spectrum of malignancies including breast cancer [2,3], pancreatic cancer [4–10], and prostate cancer [11–13]. Remarkable progress has been made in these areas with significant promise in the clinical diagnostic arena. Not only have these led to the development of novel diagnostic approaches but have also significantly impacted “molecular pharmacogenomics and therapeutics”. Several small molecule inhibitors against these markers are under development for targeted therapies in cancer treatment.

As we embark into the era of personalized medicine, using precise targets for diagnosis of cancers is important since specific drug therapies will be targeted against these molecules. Not only will these have implications in the cancer diagnosis but also in the institution of novel therapies. Treatment modalities in cancer patients are no longer based only on anatomic location and the phenotype of the tumor whether adenocarcinoma or squamous cell carcinoma. Our understanding of pathogenetic evolution of cancer has improved considerably and this has been translated into the clinical context [13]. In the clinical domain there has been a paradigm shift in the cancer diagnosis approach which has resulted in the development of novel algorithms for the therapeutic management of cancer patients that paved the way for personalized oncology.

A significant part of this is due to the understanding of alterations occurring within the cancer cells at the molecular level [4,14]. During pathogenesis and development, cancers acquire significant alterations in several cellular molecules including DNA, RNA, mRNA, miRNA and proteins. Current methodologies for cancer diagnosis have incorporated these cellular molecular changes into the cancer diagnostic realm initially at the basic research level and gradually being translated to the clinical arena.

## 2. Cancer Cell Alterations as an Aid to Cancer Diagnosis

As the tumors progress and develop invasive and metastatic capabilities, these molecular changes are deregulated due to inherent biologic properties of the cancer cell. These molecular alterations vary not only by the origin of the tumor cell but also by the degree of differentiation and invasive capacity of the cancer cell [4,11,15]. Based on *in vivo* and *in vitro* model studies conducted, it has been demonstrated that these molecular alterations play a significant role in the tumor progression as well as in the overall survival of cancer cells [11]. Additionally, these molecular alterations serve as targets to design novel therapies for cancers.

Recent research has demonstrated that these molecular markers can assist in early and accurate diagnosis and predict prognosis in cancers [4,13]. In particular, genetic and epigenetic changes in cells and high frequency of methylated genes in tumors lead to adenocarcinoma and may serve as a promising marker in the detection of cancer DNA [16,17]. A comprehensive approach based on detection of a panel of molecular alterations can give us a recognizable pattern of molecular alterations in the cancer cells which can serve as a “signature” specific for each tumor. Once such a “molecular signature” is identified in the tumor at the time of diagnosis, it can serve as a template for personalized onco-pharmacogenomics.

The rapid progress in the identification of these molecular targets in the cancer cell has led to a revolution of the “omic” in cancer diagnostics including (Whole) Genome (WGS), Exome (WES), methylome, transcriptome (including the miRnome), microbiome, metabolome, proteome and topome [18–20]. The result of this is the emergence of a new field of “Molecular Oncodiagnostics”. This has resulted in altered perspectives in the diagnostic arena [21,22]. Tumors are no more just diagnosed at the histomorphological level. Molecular alterations detected by high throughput technologies are an integral part of the diagnostic armamentarium for ultimate benefit to the patient.

## 3. Molecular Alterations for Cancer Diagnosis

**Figure 1:**
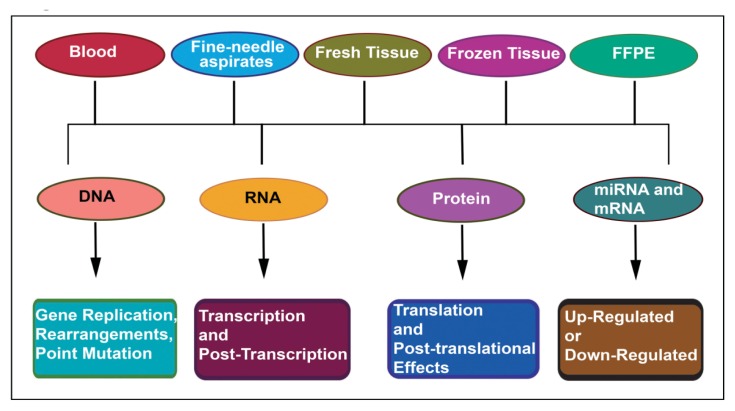
Molecular diagnostic schema representing routine biological specimens and their molecular alterations.

In the current context of the molecular alterations that have been used for the cancer diagnosis are occurring at the DNA level include gene replication, rearrangements/translocations, point mutations/deletions or insertions [13]. At the RNA level, the changes are seen in transcription and post-transcriptional modifications [4,13], and at the protein level, it is seen at the translation and post-translational effects [23,24] as depicted in Figure 1.

Recent work from our group has pioneered research in several basic and translational level for novel diagnostic approaches in cancer investigating the use of microRNAs (miRNAs) [2,4,9,12,13], which has become the focus of many recent investigations. The miRNAs are small nucleotides about 19–25 nucleotides in length, and they are non-coding endogenous single-stranded RNAs. In cancers, they are rapidly gaining importance since they actively regulate the evolution, development, progression and metastasis of cancers, and thus they provide the invasive property of cancer cells.

## 4. The miRNAs in Cancer Diagnosis

The expression levels of miRNAs are tumor specific. This property of the cancer cell is being utilized in cancer diagnosis for early and accurate cancer diagnosis [4,25,26]. Depending on their downstream signaling effect on genes and gene products, miRNAs may be up or down-regulated in cancers [14]. The ones which are up-regulated in cancers are proposed to have an oncogenic potential. Alternatively, the miRNAs which are down-regulated in malignancies are proposed to have a tumor suppressor effect. The classical examples of miRNAs with an oncogenic potential include miR-155, miR-17-92 and miR-21 and many others. The miR-21 has been found to be over-expressed in several malignancies [9,15]. The miRNAs with tumor suppressor action are the let-7 family and miR-200 family which are frequently down-regulated in many types of cancers [2,12,15,27] although there are many more miRNAs being discovered to be associated with cancers.

Previous research has demonstrated that the miRNAs regulate cancer stem cells (CSC) [12,14] and epithelial mesenchymal transition (EMT) [2,11,14] phenotype of cancer cells. CSCs are known to play a role in the development of resistance to chemotherapeutic drugs in cancers. The EMT phenomenon leads to the development of invasive characters in cancer cells leading to the acquisition of an invasive phenotype with spindled morphology [15]. Deregulation in the expression of miRNAs would thus have significant implication in the invasion, progression, developing metastatic capabilities and drug resistance in cancers [5,11,14].

## 5. Clinical Application of Cancer Diagnostic Modalities

The current oncology practice is rapidly undergoing a change. There is no more a “one size fits all” approach. Personalized treatment approaches are being developed based on the cancer diagnostic biomarkers. Translational research has shown the clinical application of the novel molecular diagnostics markers in the early diagnosis of cancer [13]. Such molecules also hold promise in risk stratification and prognosis. Before any molecule can be used in the cancer diagnosis arena, it needs to be detected in a wide variety of clinical specimens including blood [28,29], fine needle aspirates [7] and tissues both fresh frozen and paraffin embedded [13,25] as presented in Figure 1. Recent studies have demonstrated the capability of detecting these molecules in all of these clinical specimens. With the rapid evolution of molecular biology, there is a plethora of cancer diagnostic molecules with possible application in the clinical diagnostic realm. These molecules could be limited on their detection to certain patient specimens. This would greatly limit their widespread application in patient testing. On the other hand, there may be molecules like miRNAs which are detectable in a variety of patient specimens like blood, tissues and even fine needle aspirates, making them clinically applicable and potentially useful biomarkers since they can be evaluated in a variety of such patient specimens. It is the detectability of biomolecules in a variety of patient body samples that determines whether a particular biomolecule will in future have potential clinical use in the diagnostic arena.

In the current clinical context, cancer diagnosis involves the use of biomarkers examining molecules at the cellular level. Cancer diagnosis today is not just the recognition of the morphological phenotype through microscopic examination, but it is a complex blend of these microscopic features with immunohistochemical stains and molecular approaches to add value to the diagnostic report. The current standardized surgical pathology reporting formats make use of the available molecular markers to communicate prognostic and therapeutic implications with the clinicians.

Allele-specific quantitative PCR (IG/TCR-QPCR) is a robust and reproducible test with a sensitivity of at least 0.01% to detect minimal residual disease (MRD) and quantify it for widespread use in therapeutic stratification of pediatric acute lymphoblastic leukemia (ALL). It can be extended to achieve molecular MRD monitoring in adult and pediatric ALL [30]. Another test useful in the molecular monitoring of leukemia is the multiplex RT-PCR followed by liquid bead array detection. It is a rapid and flexible method which provides additional information for accurate diagnosis and prognosis [31]. These methods are easy to incorporate into the clinical laboratory workflow and generally complement the standard cytogenetic methods. Real time RT-PCR assay is currently being used in the clinical diagnostic arena for the relative quantification of the ABL1 fusion transcripts in establishing new diagnosis, determining relapse and monitoring remission by using clinical patient specimens including whole blood and bone marrows [32–34]. Further, these tests can be extended to determine the acquired mutations in the ABL1 kinase domain by RT-PCR of the ABL1 translocation which is followed by nested PCR of the ABL1 kinase domain region and bidirectional sequencing to identify mutations associated with drug resistance [35,36]. Likewise, PCR assay is also being currently clinically used to identify mutations in KRAS gene for predicting response to targeted therapies [37,38].

The microRNAs (miRNAs) are recently described emerging biomarkers for cancer diagnosis and prognosis with potential future implications in therapeutic interventions [2,3,12,13,39,40]. They have widespread clinical application due to several advantages including their application to a variety of biological specimens including blood, tissue, sputum and stool [7,41,42]. These are novel molecules since they have downstream effects on several genes and signaling pathways by their up or down regulation [43]. The biggest advantage of using the miRNAs based approaches in cancer diagnosis, prognosis and therapeutics is the ability of miRNAs to concurrently target multiple effectors of pathways involved in cell differentiation, proliferation and survival. Another advantage of using miRNAs is the fact that they are stable in body fluid and tissue samples. Recently several novel high-throughput methods have been developed for the detection of miRNAs in the cancer diagnosis and prognosis. These include electrically magnetic-controllable electrochemical biosensors [44], bead-based suspension array [45], power-free microfluidic chip [46] and one-step real time RT-PCR for detection of miRNAs [47]. For hematological malignancies, flow cytometry and molecular genomics has been an integral part of cancer diagnosis. Based on the WHO classification of hematological malignancies, genomic alterations are a composite part of the cancer diagnosis [48]. This has implications in the selection of the right treatment protocol based on these molecular changes. Further, the prognosis and risk stratification is based on these criteria.

## 6. Implications of Cancer Diagnostic Tests on Treatment

**Figure 2:**
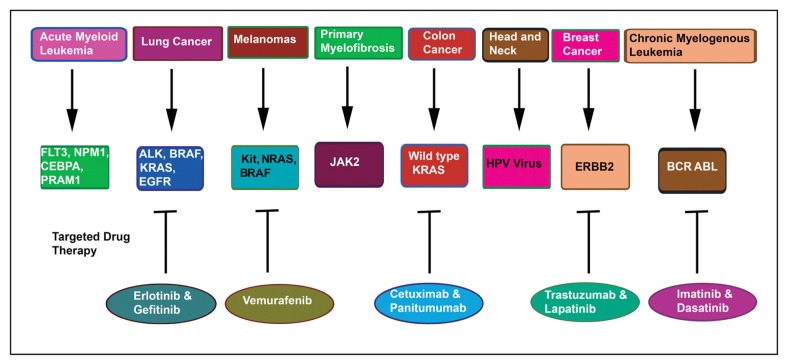
Novel diagnostic biomarkers used in the clinic for various types of cancers and their targeted drug therapy.

Several novel diagnostic biomarkers have been identified for clinical diagnosis of cancers as demonstrated in Figure 2. Some of these are also “druggable” targets against which small molecule inhibitors are under development. Each day the repertoire of targeted therapies is rapidly expanding. These include markers for hematological malignancies e.g., FLT3, NPM1, CEBPA and PRAM1 in Acute Myeloid Leukemia [48]. In myeloproliferative neoplasms like Chronic Myelogenous leukemia, BCR-ABL serves as a diagnostic marker [48]. So, are the alterations of JAK2 in Polycythemia Vera, Primary Myelofibrosis and Essential Thrombocythemia [48] has been demonstrated. Recent studies have shown the significance of ALK, EGFR, KRAS and BRAF in lung cancer [49]. BRAF, KIT and NRAS are clinically relevant in melanomas [50]. Additionally, besides tumor markers, tumor microenvironment consisting of host immune cells may control tumor behavior or function as a useful biomarker [51]. These diagnostic approaches for cancer have far reaching consequences in the therapeutic management of cancer patients. These molecules have already been incorporated in the clinical patient management for early and accurate diagnosis, determining prognosis and risk stratification of disease, as well as designing targeted molecular therapeutics.

There also has been a significant progress in the development of novel antibodies for immunohistochemical detection of proteins which is the end result of the translation due to DNA and RNA alterations that have translated from the research bench [52] and now proven to have clinical utility in the diagnosis and risk stratification of cancers [53]. This immunohistochemical detection of proteins nowadays assists in routine diagnosis in Surgical Pathology.

## 7. Novel Cancer Diagnostic Technologies

Recent advances in the field of cancer diagnosis have seen a plethora of rapid and accurate high throughput diagnostic tests. Initially used as research tools [2,3,6,11,12,54], these molecular diagnostic tests have now been demonstrated to be applicable in the clinical scenario [4,7,13]. The use of these biomarkers in cancer diagnosis has been facilitated by the availability of several high throughput and high resolution technologies for the detection of these novel biomarker abnormalities as presented in Table 1. Based on basic, translational and clinical research new platforms like qualitative PCR-ARMS and RFLP, real time PCR-TaqMan assays, nested PCR, FISH, capillary electrophoresis, sequencing/pyrosequencing, sequenom [55], targeted gene panel sequencing and microarrays [13] are available for clinical use in cancer diagnosis.

Quantitative PCR has widespread application in the detection of DNA/RNA/miRNA abnormalities for initial diagnosis of cancer. These would also be applicable in surveillance, follow-up and monitoring treatment outcome of cancer patient. It can be used for single-nucleotide polymorphism (SNP) detection, gene expression profiling and also quantification of viral load in infection associated cancers. Gel electrophoresis has its limitations including low resolution, poor precision, and results not being quantifiable. Hence, capillary electrophoresis has been developed with widespread application in detecting gene rearrangements, single nucleotide polymorphism (SNP), and loss of heterozygosity (LOH) [56]. With the availability of sequencing and rapid technological advances, the cost of performing sequencing for cancer diagnosis with >46 gene panels is about $1000, and the turn-around time is now 48 h [57].

The sub-cellular deciphering of molecular alterations can be used as tumor specific “signatures” for cancer diagnosis. Targeted diagnostic strategies using DNA, RNA, miRNA or proteomic approaches have enabled early and accurate diagnosis. This varies from a “single-gene” diagnosis to a panel of tumor specific alterations used to identify a tumor phenotype. Incorporation of bioinformatics has enabled evaluation of multiple genes and miRNAs in parallel for identifying clinical algorithms for cancer diagnosis [13].

## 8. Molecular Cancer Diagnosis Panels

A typical example of a genomic panel recommended for cancer diagnosis is a panel comprising of BRAF, KIT, NRAS, GNA11 and GNAQ for Melanomas [50]. For lung cancers, the genomic panel comprises of ALK, EGFR, KRAS and BRAF [49]. For acute myeloid leukemia the panel is composed of FLT3ITD/FLT3-TKD, NPM1, C-KIT, PTPN11 and CEBPA [48].

Upon diagnosis of rearrangement of ALK gene in NSCLC lung cancers, drugs have been approved for clinical use. However with the detection of KRAS mutation in lung cancers the drugs are currently under development. With the use of recent high throughput technologies like the Sequenom Maldi-TOF spectrometry gene panels can be evaluated for diagnosis of cancers [48,58]. These studies allow multiplexing with up to 15 patients and 11 genes in two days. Apart from genetic alterations for assisting the cancer diagnosis, these methodologies can also be used for SNP genotyping, methylation assay and even quantitative gene expression analysis [58].

The molecular targets identified for cancer diagnosis have implications in the treatment protocols. This has led to the development of “pharmacogenomics” and “pharmacogenetics” [62] which are the central component of personalized medicine. It has also contributed in identifying variants which may influence drug metabolism or interaction of a drug with its cellular target, allowing customization of choice of drugs and dosage. In the clinical arena, its objectives are to avoid adverse drug reactions, maximize drug efficacy and to select responsive patients [62].

In cancer diagnosis, the role of the Pathologist is to link the phenotypic expression of the tumor visualized microscopically with the variation in molecular genotype. For personalized medicine the genetic alterations occurring in cancers are used not only for diagnosis but also for targeted drug therapy as displayed in Figure 2. The typical examples include BCR-ABL, a target for Imatinib and Dasatinib therapy in Chronic Myeloid Leukemia [63]. In melanomas, the BRAF mutations are targeted with Vemurafenib [64]. In non-small cell lung cancers (NSCLC), EGFR mutations are targeted with Gefitinib and Erlotinib [65]. Cetuximab and Panitumumab are targeted against wild-type KRAS in colon cancers [66]. Several recent reports suggested that aspirin usage improves colon cancer mortalities [67–69] especially among patients with mutated-PIK3CA, indicating PIK3CA as potential biomarker in colon cancer [68,69]. The second major genomic instability pathway involved in pathogenesis of colon cancer is the microsatellite instability (MSI) pathway caused by mutations in the DNA mismatch repair genes (MMR). Deficiency in MMR genes in tumors has been related to improved prognosis, and thus represents one of the most promising molecular markers for chemo-sensitivity [70,71]. ERBB2 amplification in breast cancers is targeted with Trastuzumab and Lapatinib [72] but these are only some recent examples.

To assist the diagnosis of cancers, there are commercially available cancer panels like the Oncocarta [13] and the Ampliseq cancer panels [60]. The Ampliseq is a panel comprising of 739n hotspot cancer mutations in 46 genes involving hematological and solid tumor cancers. The recent application of microarray technology to analyze the expression analysis of more than 1000 miRNAs simultaneously has helped in the diagnosis of multiple cancers [7,12,13]. In Oncotype Dx 21 genes are profiled based on which the recurrence risk is predicted at the time of diagnosis [73]. The MammaPrint is a 70 gene microarray panel for risk stratification [59]. In both Oncotype Dx and MammaPrint, the patients with low score are given only hormonal therapy and high risk ones are also given chemotherapy. Afirma gene expression profiling is used in the classification of thyroid nodules [61]. It sub classifies cases which need further surgery if suspicious on fine-needle aspirates (FNA).

Deep understanding of the pathogenesis of cancer has led to unearthing of novel molecular diagnostic tools for cancer diagnosis. High throughput technology of molecular genetics and genomic analysis of sub-cellular molecules has rapidly changed the diagnostic landscape of cancer testing [13]. High-resolution molecular cytogenetic analysis has enables detection of deletions and duplications of DNA, RNA and miRNA, well below the resolution of the light microscope. Diagnostic testing for “single-gene” disorders can be done by targeted analysis for specific mutations, by sequencing a specific gene to scan for mutations, or by analyzing multiple genes in which mutation may lead to a similar phenotype [13]. The advent of massively parallel next-generation sequencing facilitates the analysis of multiple genes and now is being used to sequence the coding regions of the genome (the exome) for clinical testing. Exome sequencing requires bioinformatics analysis of the thousands of variants that are identified to find one that is contributing to the pathology; there is also a possibility of incidental identification of other medically significant variants, which may complicate genetic counseling. DNA testing can also be used to identify variants that influence drug metabolism or interaction of a drug with its cellular target, allowing customization of choice of drugs and dosage. Exome and genome sequencing are being applied to identify specific gene changes in cancer cells to guide therapy, to identify inherited cancer risk, and to estimate prognosis. Genomic testing may be used to identify risk factors for common disorders, although the clinical utility of such testing is unclear. Genetic and genomic tests may raise new ethical, legal, and social issues, some of which may be addressed by existing genetic nondiscrimination legislation, but which also must be addressed in the course of genetic counseling.

Advances in molecular microbiology have also had far reaching consequences and impact on cancer diagnosis. This includes the detection of viruses in cancers which have prognostic implications. A typical example of this is the utilization of the HPV detection in head and neck cancers which assist in the risk stratification of cases [74]. In future, these molecular features would become an integral part of all cancer diagnosis with implications in treatment management protocols. Most hematological malignancies have already seen that trend and solid tumors are following the same route, which would likely revolutionize cancer diagnosis and personalized therapy.

## 9. Conclusions

In conclusion, the diagnosis of cancer has undergone a paradigm shift. No longer is cancer diagnosed only based on morphological parameters. More and more the diagnostic algorithm is supported by immunohistochemical and molecular alterations at the DNA, mRNAs, miRNAs and proteomic level. Multiple platforms and high throughput technological advances enable faster and cheaper analysis of all these as well as the whole genome. This is having a significant impact on how medicine is now being practiced in a personalized approach leading to the development of precision medicine based on pharmacogenomics. It is being realized that a tumor may not be characterized by a single gene alteration but by a panel of ‘signature’ genomic alterations leading to targeted therapeutic strategies and surveillance based on the tumor specific alterations. The ultimate goal of cancer diagnosis in personalized medicine would be to identify the correct diagnosis and guide the therapy so that every patient received precision medicine that is the right drug at the right dose.

## Figures and Tables

**Table 1:** Current high throughput tests for cancer diagnosis.

Analysis	Method	References
MicroRNA and RNA	Microarray technology	[13]
Single nucleotide polymorphism (SNP) arrays, gene arrangements	Capillary electrophoresis	[56]
Single nucleotide polymorphism genotyping	Matrix-associated laser desorption/ionization time-of-flight mass spectrometry (MALDI-TOF MS) sequenom	[58]
Methylation analysis	Quantitative sequenom and pyrosequencing	[55]
70 Gene microarray panel analysis in breast cancer	MammaPrint	[59]
Hotspot cancer mutations	Ampliseq	[60]
Classification of thyroid nodules	Afirma gene profiling	[61]
